# Global, regional and national burdens of gout in the young population from 1990 to 2019: a population-based study

**DOI:** 10.1136/rmdopen-2023-003025

**Published:** 2023-04-24

**Authors:** Jing Zhang, Chenye Jin, Bing Ma, Hao Sun, Yanmei Chen, Ying Zhong, Cheng Han, Tingting Liu, Yongze Li

**Affiliations:** 1 Department of Biostatistics and Epidemiology, School of Public Health, Health Science Center, Shenzhen University, Shenzhen, China; 2 Department of Rheumatology and Immunology, The First Hospital of China Medical University, Shenyang, China; 3 Department of Clinical Epidemiology and Evidence-based Medicine, The First Hospital of China Medical University, Shenyang, China; 4 School of Mathematics and Systems Science, Guangdong Polytechnic Normal University, Guangzhou, China; 5 Department of Pediatrics, Shenzhen United Family Hospital, Shenzhen, China; 6 Department of Clinical Nutrition and Metabolism, Affiliated Zhongshan Hospital of Dalian University, Dalian, China; 7 Department of Endocrinology and Metabolism, The Institute of Endocrinology, NHC Key Laboratory of Diagnosis and Treatment of Thyroid Disease, The First Hospital of China Medical University, Shenyang, Liaoning, China; 8 Department of Epidemiology, School of Public Health, China Medical University, Shenyang, China

**Keywords:** gout, epidemiology, outcome assessment, health care

## Abstract

**Objective:**

To use data from the Global Burden of Disease (GBD) Study 2019 to report the global, regional and national rates and trends of annual incidence, point prevalence and years lived with disability (YLD) for gout in adolescents and young adults aged 15–39 years.

**Methods:**

We conducted a serial cross-sectional study of gout burden in the young population aged 15–39 years using data from GBD Study 2019. We extracted rates per 100 000 population of incidence, prevalence and YLD of gout, then calculated their average annual percentage changes (AAPCs) at the global, regional and national level between 1990 and 2019 by sociodemographic index (SDI).

**Results:**

The global gout prevalent cases in individuals aged 15–39 years was 5.21 million in 2019, with the annual incidence substantially increasing from 38.71 to 45.94 per 100 000 population during 1990–2019 (AAPC 0.61, 95% CI 0.57 to 0.65). This substantial increase was observed in all SDI quintiles (low, low-middle, middle, high-middle and high) and every age subgroup (15–19, 20–24, 25–29, 30–34 and 35–39 years). Males accounted for 80% of the gout burden. High-income North America and East Asia were facing a substantial increase in gout incidence and YLD simultaneously. Elimination of high body mass index can reduce 31.74% of the gout YLD globally in 2019, which varied from 6.97% to 59.31% regionally and nationally.

**Conclusion:**

Gout incidence and YLD in the young population grew simultaneously and substantially in both developed and developing countries. Improving representative national-level data on gout, interventions for obesity and awareness in young populations are strongly suggested.

**WHAT IS ALREADY KNOWN ON THIS TOPIC**

- Gout prevalence increased globally in the general population between 1990 and 2019.
- Most of the gout burden was in the elderly population and developed countries.
- Less than half of patients with gout started urate-lowering therapy, among which less than half adhered to this therapy.

**WHAT THIS STUDY ADDS**

- This is the first comprehensive report on the trends of gout incidence, prevalence and years lived with disability (YLD) among adolescents and young adults aged 15–39 years at the global, regional and national levels from 1990 to 2019.
- It reports the incidence, prevalence and YLD by sex, age and a country’s sociodemographic index (SDI) at the global level.
- It quantified the fraction of risk factors that contributed to the gout YLD among this population.

**HOW THIS STUDY MIGHT AFFECT RESEARCH, PRACTICE OR POLICY**

- The global gout incidence and YLD among individuals aged 15–39 years increased simultaneously and substantially within all five SDI quintiles and every age subgroup from 1990 to 2019.
- Controlling high body mass index can reduce one-third of gout YLD globally, and even half in certain regions and countries.
- This study advocates for national-level data and awareness of gout intervention and treatment in adolescents and young adults globally.

## Introduction

Gout is the most common form of inflammatory arthritis globally, lasting days to weeks if untreated and resulting in hospitalisations, intense pain, work absence, erectile dysfunction and even subsequent cardiovascular events.^1–6^

According to the previous gout study of Global Burden of Disease (GBD) 2017, the gout burden was rising in the general population across the globe, while the suboptimal management of gout continues worldwide.^7^ According to recent evidence of GBD 2019, a decreasing trend in disability-adjusted life years of musculoskeletal disorders was mainly driven by the decrease in low back pain, while the increasing trend was largely due to gout across age groups, which was left unknown.^8^

Previous studies mainly focus on the gout burden in the population aged above 40 years and in developed regions and countries.^7 9 10^ Gout in the young population was generally ignored owing to the lack of national-level data in most countries, especially in developing countries.^11–14^ Moreover, being at a young age could decrease adherence to gout treatment and management, leading to a higher health burden. It is urgent to understand the incidence, prevalence, years lived with disability (YLD) and trends among the young population in recent years worldwide.

In this study, we hypothesised an increase in gout annual incidence, point prevalence and YLD in youth and young adults under 40 years of age between 1990 and 2019. We aimed to examine the global trends of the above rates since 1990; to stratify the global trends by age group, sex and sociodemographic index (SDI); to report the above rates at the regional and national levels; and to clarify the risk factor of the YLD from gout.

## Methods

### Study population and data collection

In this analysis of the GBD Study 2019, we obtained repeated cross-sectional data from the Global Health Data Exchange, which includes the global burden of 369 diseases and injuries, including gout, and 84 risk factors, including high body mass index (BMI) and kidney dysfunction, in 21 regions and 204 countries and territories from 1990 to 2019. The data of GBD are identified from a systematic review of published studies, searches of government and international organisation websites, published reports, primary data sources such as the Demographic and Health Surveys, and contributions of datasets by GBD collaborators to ensure the quality of the surveys. More detailed protocol of GBD is described online (http://www.healthdata.org/gbd/about/protocol). The above gathered data were processed through standardised steps including input data, age–sex splitting, cause aggregation and noise reduction, then analysed by major models including the cause of death ensemble model, spatiotemporal Gaussian process regression and DisMod-MR. Details of the methodology used in GBD 2109 have been explained in previous studies^15 16^ and are presented in online supplemental data 2. The Global Burden of Disease Study adheres to the guidelines for accurate and transparent health estimates reporting statement. The Institute for Health Metrics and Evaluation (IHME), the institute responsible for administering the Global Burden of Disease, uses only de-identified and aggregated data.

10.1136/rmdopen-2023-003025.supp1 Supplementary data

GBD 2019 uses the case definition of primary gout given by the American College of Rheumatology 1977 survey criteria requiring the presence of monosodium urate (MSU) crystals in joint fluid or the presence of a tophus proven to contain MSU crystals and at least 6 of 12 gout symptoms or findings (>1 attack of acute arthritis, development of maximal inflammation within a day, attack of monoarticular arthritis, observation of joint erythema, pain or swelling in the first MTP joint, a unilateral attack involving the first MTP joint, unilateral attack involving tarsal joint, suspected tophus, hyperuricaemia, asymmetrical swelling within a joint on X-ray and negative culture of joint fluid for microorganisms during the attack of joint inflammation) to make a diagnosis. Gout was modelled to be non-fatal in GBD 2019. The input data and methodological summary for gout can be found in online supplemental data 3.

In this study, youth and young adults represent a heterogeneous population consisting of individuals aged 15 to 39 years. In GBD 2019, it was also assumed that there was no case in which a diagnosis occurred before the age of 15 years. Data were collected on the above indicators of gout from both sexes, in five age groups (15–19 years, 20–24 years, 25–29 years, 30–34 years and 35–39 years), and by the 21 regional groupings of countries that are geographically close and epidemiologically similar, as defined in the GBD project. GBD 2019 also calculates the SDI of each country, which is a composite indicator of social and economic conditions that influence health outcomes in each location. It is calculated as the geometric mean of 0 to 1 indices of total fertility rate for individuals younger than 25 years, the mean number of years of education for individuals 15 years old and older, and lag distributed income per capita, in which 0 represents the fewest years of education, the lowest per capita income and the highest fertility rate. The SDI has five quintiles: low, low-middle, middle, high-middle and high.

### Statistical analysis

The numbers of incident cases, prevalent cases, and YLD incidence and prevalence were extracted directly from GBD 2019. All rates are reported per 100 000 population. The 95% uncertainty intervals (UIs) were defined by the 25th and 975th values of the ordered 1000 estimates based on GBD’s algorithm. Global trends of gout incidence, prevalence and YLD were assessed. We calculated the age-specific rates and their average annual percentage changes (AAPCs) using linear regression with rates on the logarithmic scale as the dependent variable and each year as the independent variable. The AAPC is a summary measure of the trend over a prespecified fixed interval and is computed as a weighted average of the annual percentage change (APC), allowing us to use a single number to describe the average APCs over a period of multiple years. The APC was calculated using the geometrically weighted average of the various annual percentage change values in the regression analysis. The value of the AAPC indicates how many percentages change annually (increase, decrease or no change). For example, if the AAPC was 0.1, then it would suggest an annual rate increase of 0.1%. The age-specific rates and cases and their 95% UIs were directly obtained from the IHME website. All estimates were generated from the mean of 1000 draws, and 95% UIs were determined using the 2.5th and 97.5th centiles of the ordered draws according to the GBD methodology.^15^ The trends of rates were reflected in AAPC values and their 95% CIs. We calculated the AAPCs between 1990 and 2019.

The proportional attributable fraction was calculated as a proportional reduction in gout, which would occur if exposure to these risk factors was reduced to the theoretical minimum risk exposure level. In GBD 2019, high BMI and impaired kidney function were identified as risk factors associated with the onset of gout. The definitions and prevalence of these two risk factors and their relative risks for gout have been well described in a previous study, along with the appropriate methods.^17^ High BMI was defined as BMI ≥25 kg/m^2^ for individuals aged ≥20 years, and thresholds from the International Obesity Task Force standards were used for individuals aged <20 years.^18^ Detailed information about the process of data selection and data inputs has been published previously.^18^

## Results

### Global trends

**Figure 1:**
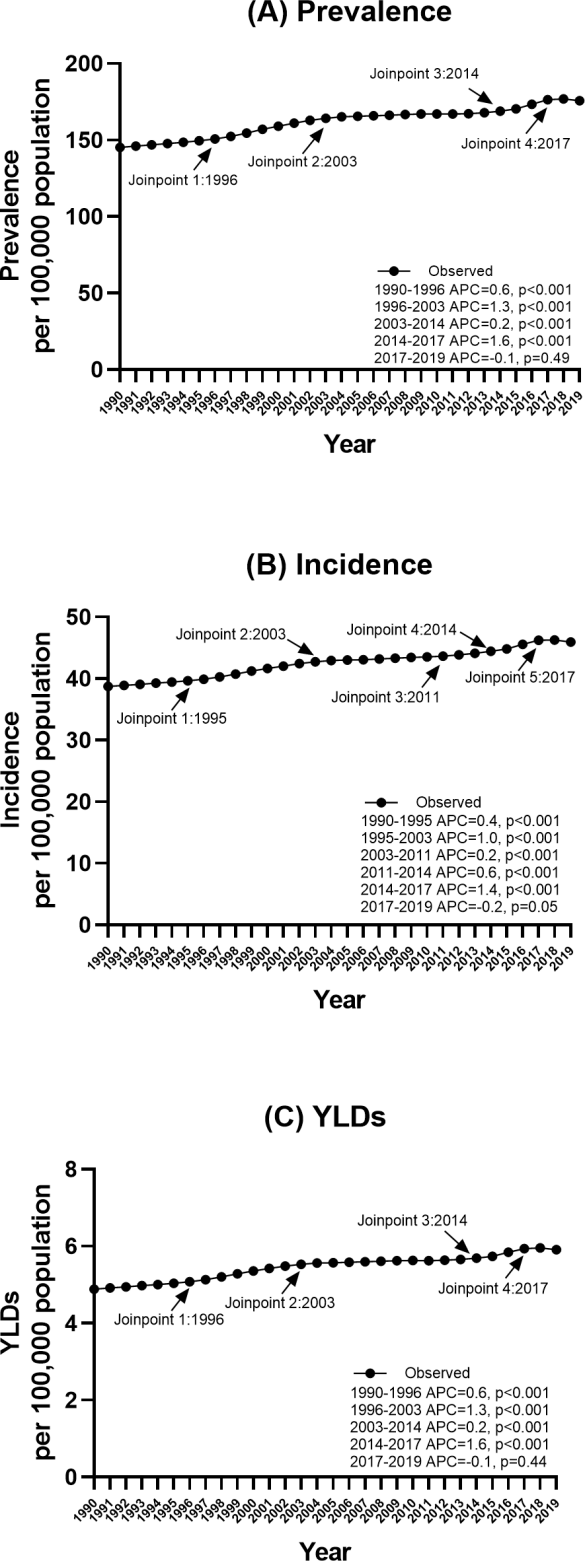
Joinpoint regression analysis of the prevalence, incidence and years lived with disability (YLD) for gout globally in individuals aged 15–39 years from 1990 to 2019. (A) Prevalence; (B) incidence; (C) YLDs.

Globally, the gout incidence in individuals aged 15–39 years increased between 1990 and 2019 (from 38.71 per 100 000 population [95% UI 27.10 to 53.46] in 1990 to 45.94 per 100 000 population [32.26 to 63.29] in 2019; AAPC 0.61 [95% CI 0.57 to 0.65]). Accordingly, the gout prevalence also increased between 1990 and 2019 (from 145.19 per 100 000 population [100.20 to 202.76] in 1990 to 175.68 per 100 000 population [123.78 to 242.53] in 2019; AAPC 0.7 [95% CI 0.60 to 0.73]). YLD increased between 1990 and 2019 (from 4.89 per 100 000 population [95% UI 2.91 to 7.75] in 1990 to 5.91 per 100 000 population [3.59 to 9.14]; AAPC 0.66 [95% CI 0.60 to 0.73]). Overall, the number of gout annual incident cases, point prevalent cases and YLD in the younger population in 2019 was 1.6 times that in 1990 (incident cases: 1 363 085 in 2019 vs 849 175 in 1990; prevalent cases: 5 213 860 in 2019 vs 3 184 743 in 1990; YLD: 175 377 in 2019 vs 107 156 in 1990) (online supplemental data 1). The gout prevalence, incidence and YLD in each year between 1990 and 2019 can be found in online supplemental data 4. During the same period, the AAPC of gout incidence among the senior population aged over 55 years was 0.93 (95% CI 0.83 to 1.03) (online supplemental data 5). The gout incidence substantially increased in 1996, 2003 and 2011, and had its largest increase in 2014–2017, with an AAPC of 1.38, followed by a statistically non-significant decrease in 2017–2019, with an AAPC of −0.23 (figure 1 and online supplemental data 6). Gout prevalence and YLD followed the same pattern.

In 2019, there were 1.36 million gout incident cases, 5.21 million prevalent cases and 0.18 million YLD among individuals aged 15–39 years, accounting for 14.78% of the 9.22 million gout incident cases, 9.68% of the 53.87 million gout prevalent cases and 10.48% of the 1.67 million YLD among all populations globally.

### Global trends by sex

There were global increases in gout incidence from 1990 to 2019 in both males and females, with an AAPC of 0.63 (95% CI 0.58 to 0.68; from 61.57 per 100 000 population [95% UI 43.57 to 85.02] in 1990 to 73.59 per 100 000 population [52.08 to 101.16] in 2019) in males and an AAPC of 0.51 (95% CI 0.48 to 0.54; from 15.31 per 100 000 population [95% UI 10.17 to 21.80] to 17.56 per 100 000 population [11.83 to 25.03]) in females. Accordingly, the gout prevalence also increased between 1990 and 2019 in both males (AAPC 0.69 [95% CI 0.61 to 0.77]) and females (AAPC 0.54 [95% CI 0.51 to 0.58]). There were also global increases in gout YLD from 1990 to 2019 in both males and females, with an AAPC of 0.69 (95% CI 0.61 to 0.77; from 7.73 per 100 000 population [95% UI 4.65 to 12.12] in 1990 to 9.45 per 100 000 population [5.74 to 14.41] in 2019) in males and 0.54 (95% CI 0.51 to 0.58; from 1.97 per 100 000 population [95% UI 1.12 to 3.17] to 2.27 per 100 000 population [1.30 to 3.66]) in females (table 1).

In 2019, males accounted for 1.11 million (81.12%) of gout incident cases, 4.23 million (81.21%) of prevalent cases and 0.14 million (81.0%) of gout YLD among the young population globally (online supplemental data 1).

### Global trends by age group

Globally, the largest increase in gout incidence between 1990 and 2019 was observed in those aged 35–39 years with an AAPC of 0.47 (95% CI 0.40 to 0.55; from 96.09 per 100 000 population [95% UI 57.25 to 153.22] in 1990 to 105.09 per 100 000 population [62.45 to 166.08] in 2019), followed by those aged 30–34 years (AAPC 0.40 [95% CI 0.35 to 0.45]), aged 25–29 years (AAPC 0.33 [95% CI 0.28 to 0.37]), aged 20–24 years (AAPC 0.29 [95% CI 0.21 to 0.36]) and aged 15–19 years (AAPC 0.17 [95% CI 0.11 to 0.22]). The increasing trends of YLD followed the same pattern (table 1).

In 2019, those aged 35–39 years, 30–34 years and 25–29 years accounted for 0.57 million (41.70%), 0.43 million (31.40%) and 0.25 million (18.10%) of the incidence of gout cases among individuals, respectively; 2.53 million (48.58%), 1.66 million (31.80%) and 0.78 million (14.97%) of the prevalent gout cases, respectively; and 84 213 (48.02%), 55 995 (31.93%) and 26 654 (15.20%) of the YLD cases, respectively (table 1).

### Global trends by SDI

Globally, all countries with different SDI quintiles had a substantial increase in gout incidence among individuals aged 15–39 years. The largest increase in gout incidence by SDI quintile was observed in the high-SDI quintile with AAPC of 1.28 (95% CI 1.26 to 1.29; from 53.39 per 100 000 population [95% UI 37.34 to 74.72] in 1990 to 77.65 per 100 000 population [56.70 to 103.36] in 2019), followed by high-middle SDI quintiles (AAPC 1.1 [95% CI 1.03 to 1.17]), middle-SDI quintiles (AAPC 0.71 [95% CI 0.63 to 0.79]), low-middle SDI quintiles (AAPC 0.39 [95% CI 0.34 to 0.45]) and low-SDI quintiles (AAPC 0.25 [95% CI 0.23 to 0.26]). The increasing rate of gout incidence in the high-SDI quintile was more than five times the rate in the low-SDI quintile. The trends in gout prevalence followed the same pattern. Accordingly, all countries with different SDI quintiles had a substantial increase in gout YLD, with the largest increase observed in the high-SDI quintile, with an AAPC of 1.4 (95% CI 1.35 to 1.44; from 7.29 per 100 000 population [95% UI 4.39 to 11.53] in 1990 to 11.01 per 100 000 population [95% UI 6.95 to 16.24] in 2019), followed by the high-middle SDI quintile (AAPC 1.15 [95% CI 1.08 to 1.21]), middle-SDI quintiles (AAPC 0.78 [95% CI 0.67 to 0.90]), low-middle SDI quintiles (AAPC 0.45 [95% CI 0.38 to 0.52]) and low-SDI quintiles (AAPC 0.27 [95% CI 0.25 to 0.29]) (table 1).

In 2019, the high-SDI quintiles had the highest gout incidence, prevalence and YLD rate, but the middle-SDI quintiles had the largest number of gout incident cases, prevalent cases and YLD, accounting for 0.45 million (33.61%), 1.71 million (32.88%) and 57 896 (33.01%), respectively (online supplemental data 7).

### Regional trends

At the regional level, the largest increases in gout incidence between 1990 and 2019 were observed in high-income North America (from 77.4 per 100 000 population [95% UI 54.02 to 108.84] in 1990 to 122.72 per 100 000 population [91.52 to 159.93] in 2019; AAPC 1.64 [95% CI 1.58 to 1.70]), East Asia (from 55.46 per 100 000 population [95% UI 39.32 to 76.44] in 1990 to 80.07 per 100 000 population [56.28 to 111.59] in 2019; AAPC 1.36 [95% CI 1.24 to 1.48]) and Tropical Latin America (from 20.74 per 100 000 population [95% UI 14.06 to 28.90] in 1990 to 28.75 per 100 000 population [19.90 to 40.16] in 2019; AAPC 1.17 [95% CI 1.14 to 1.21]). The largest increase in gout YLD between 1990 and 2019 was observed in high-income North America (from 11.06 per 100 000 population [95% UI 6.65 to 17.54] in 1990 to 18.15 per 100 000 population [11.67 to 26.07] in 2019; AAPC 1.74 [95% CI 1.66 to 1.82]), East Asia (from 6.94 per 100 000 population [95% UI 4.15 to 10.94] in 1990 to 10.33 per 100 000 population [6.08 to 16.19] in 2019; AAPC 1.45 [95% CI 1.28 to 1.62]) and Australasia (from 6.91 per 100 000 population [95% UI 4.08 to 11.29] in 1990 to 10.04 per 100 000 population [5.91 to 15.70] in 2019; AAPC 1.21 [95% CI 1.28 to 1.62]) (table 1).

In 2019, the highest gout incidence and YLD rate were observed in high-income North America, but East Asia had the largest number of gout incidents and YLD cases, accounting for 0.41 million (30.29%) of the incident gout cases and 53 247 (30.36%) of the YLD worldwide.

### National trends

**Figure 2:**
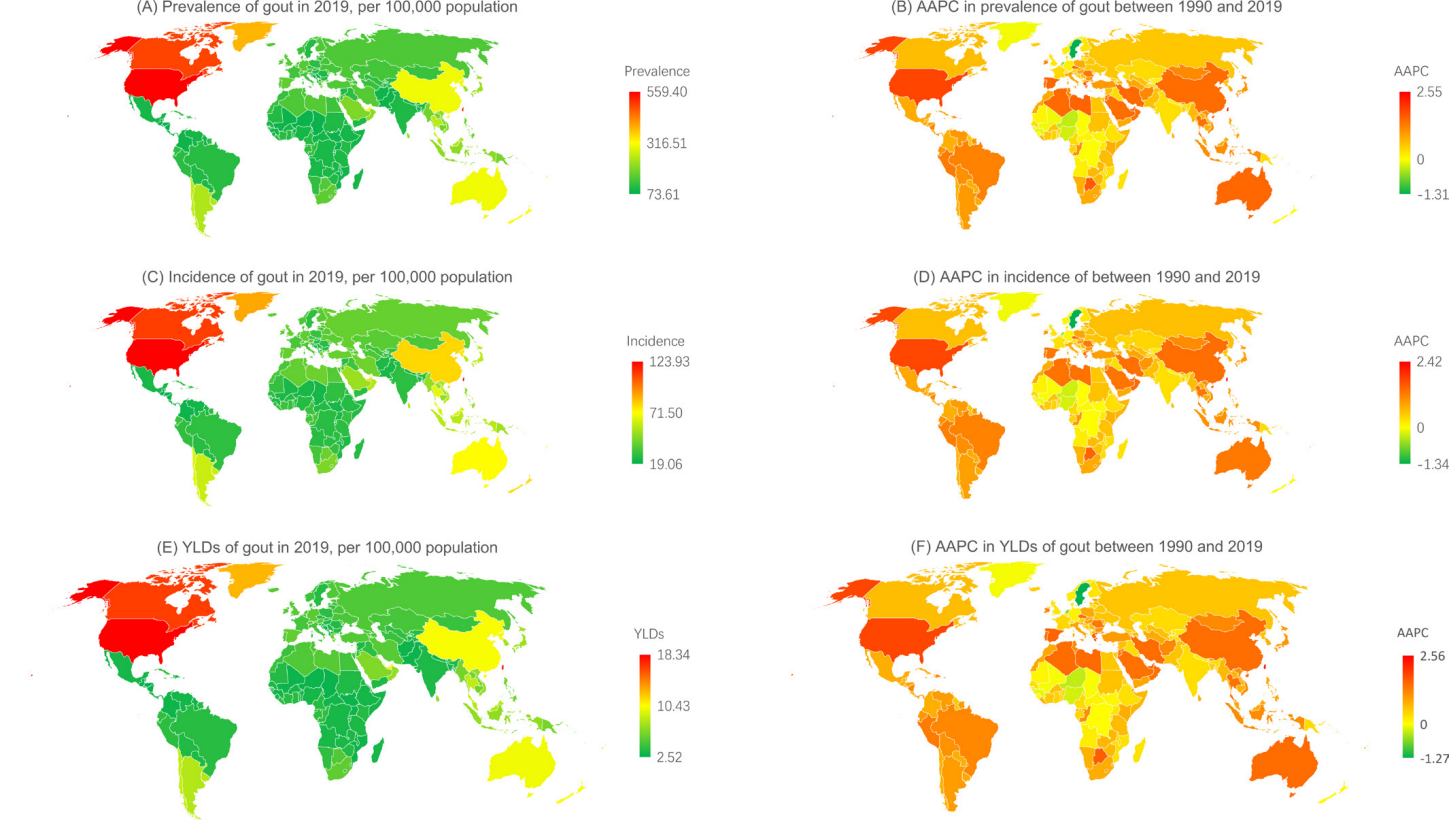
Global map of rates of gout in 2019 and their average annual percentage changes (AAPCs) from 1990 to 2019. (A) Prevalence of gout in 2019; (B) average annual percentage change in prevalence of gout between 1990 and 2019; (C) incidence of gout in 2019; (D) AAPC in incidence of gout between 1990 and 2019; (E) years lived with disability (YLDs) from gout in 2019; (F) average annual percentage change in YLDs from gout between 1990 and 2019.

At the national level, the most pronounced increase in the incidence of gout between 1990 and 2019 was in the Maldives (from 39.14 per 100 000 population [95% UI 27.06 to 54.07] in 1990 to 84.00 per 100 000 population [59.25 to 120.38]; AAPC 2.42 [95% CI 1.93 to 2.90]), USA (from 76.34 per 100 000 population [95% UI 53.01 to 106.90] in 1990 to 123.93 per 100 000 population [92.28 to 160.63]; AAPC 1.75 [95% CI 1.68 to 1.83]) and Taiwan Province of China (from 75.52 per 100 000 population [95% UI 54.17 to 104.64] in 1990 to 110.06 per 100 000 population [95% UI 79.37 to 151.06] in 2019; AAPC 1.50 [95% CI 0.95 to 2.04]). The most pronounced increase in gout prevalence and YLD between 1990 and 2019 were also found in these same three countries (figure 2).

In 2019, China, India and the USA had the largest number of gout incident cases and YLD, accounting for 0.40 million (29.09%), 0.17 million (12.34%) and 0.14 million (10.00%) of the global gout incident cases, respectively, and 50 947 (29.04%), 20 514 (11.70%) and 20 170 (11.50%) of the global gout YLD cases, respectively (figure 2).

### Risk factors

**Figure 3:**
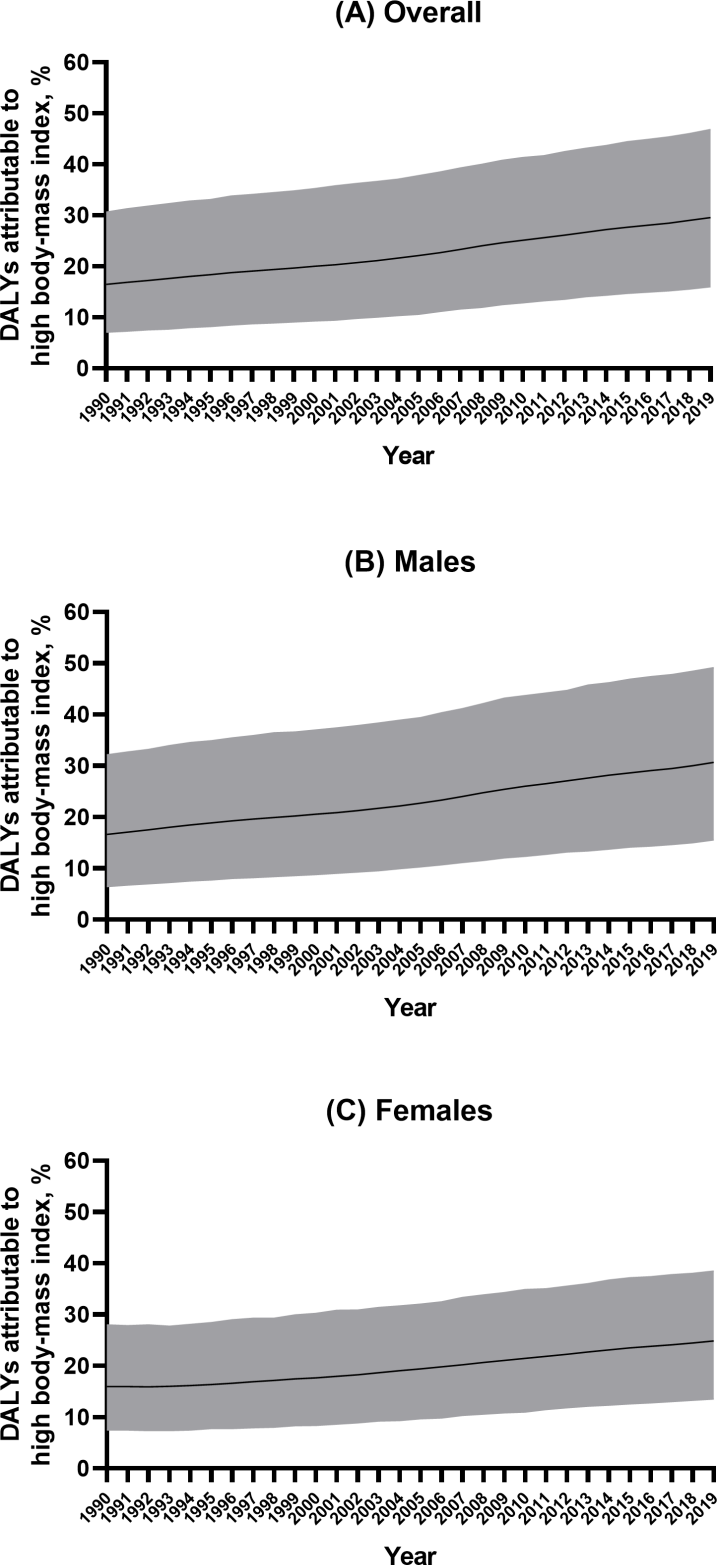
Percentage of years lived with disability due to gout attributable to risk factors in the Global Burden of Disease study in 2019, according to sex. (A) Overall; (B) males; (C) females. DALY, disability-adjusted life year.

**Figure 4:**
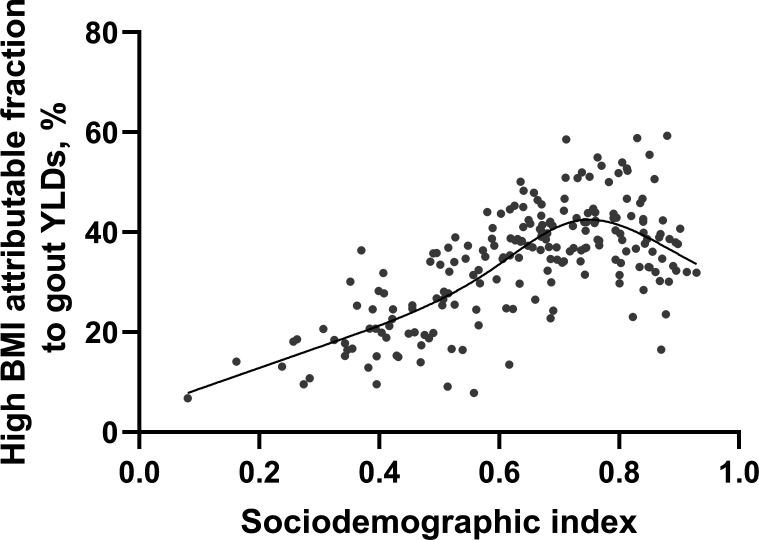
Years lived with disability (YLD) from gout per 100 000 population by 204 countries and sociodemographic index, 2019. Each point shows observed YLD for each country in 2019. BMI, body mass index.

Globally, in 2019, high BMI and kidney dysfunction accounted for 29.59% of YLD (95% UI 16.00% to 46.82%) and 1.45% of YLD (95% UI 1.22% to 1.71%), respectively. YLD attributable to high BMI was higher in males (30.69%, 95% UI 15.56% to 49.10%) than in females (24.88%, 95% UI 13.55% to 38.49%) (figure 3). The YLD attributable to high BMI increased with age and reached its highest level in the 35–39 age groups among young adults (32.04%, 95% UI 17.68% to 50.11%). The regional attributable risk fraction of high BMI ranged from 49.93% to 7.77%, with the highest observed in high-income North America (49.93%, 95% UI 28.93% to 70.92%), Australasia (46.09%, 95% UI 27.32% to 66.82%), and North Africa and the Middle East (43.31%, 95% UI 26.60% to 61.69%). (More data can be found in online supplemental data 8.) The national attributable risk fraction of high BMI ranged from 59.31% to 6.79%, with the highest observed in the United Arab Emirates (59.31%, 95% UI 37.87% to 77.64%), Qatar (58.83%, 95% UI 37.13% to 78.45%) and American Samoa (58.57%, 95% UI 40.03% to 75.77%), with the lowest observed in Somalia (6.79%, 95% UI 1.43% to 16.95%), the Democratic People’s Republic of Korea (7.82%, 95% UI 1.31% to 20.53%) and Timor-Leste (9.12%, 95% UI 2.89% to 20.55%) (figure 4).

The YLD attributable to kidney dysfunction was higher in females (1.61%, 95% UI 1.34% to 1.92%) than in males (1.40%, 95% UI 1.18% to 1.67%). The regions with the highest renal dysfunction attributable burden of gout were Central Asia (2.97%, 95% UI 2.48% to 3.53%), Central Latin America (2.88%, 95% UI 2.41% to 3.44%) and Eastern Europe (2.72%, 95% UI 2.22% to 3.31%). (More data can be found in online supplemental data 9.)

## Discussion

To our knowledge, this is the first study to describe the gout burden and its changing trends among individuals aged 15–39 years from 1990 to 2019 at the global, regional and national levels, by age, sex and SDI. The global annual incidence, point prevalence and YLD of gout in this young population have increased substantially in the past three decades, reaching 1.36 million incident cases, 5.21 million prevalent cases and 0.18 million YLD in 2019. The young population in all five SDI quintiles in all age subgroups for both sexes had an increasing gout burden. Globally, high BMI contributed to 30% of YLD from gout among the young population; regionally, high BMI contributed to nearly 50% of YLD in high-income North America, Australasia, North Africa and the Middle East.

The increasing speed of gout incidence among the young population was nearly two-thirds the speed among the senior population aged over 55 years, who were the focus of gout burden control in previous studies. This comparison indicates that urgent attention on gout burden control among the young population aged 15–39 years was urgently required. The simultaneous increase in incidence and YLD suggested that the prevention and treatment targeted at young patients with gout were highly unsuccessful. According to previous studies in the general population with gout, less than 30% of gout patients started urate-lowering therapy (ULT) within 12 months of diagnosis,^19^ among which less than 50% maintained adherence to ULT beyond a year.^20^ However, being at a younger age decreased both the adherence to and persistence with ULT. Since gout is not curable but manageable, the early onset of gout in the young population could considerably add to YLD, healthcare costs related to frequent emergency room visits^5^ and loss of work productivity,^21^ although gout is preventable, especially among the young population.^22^ Considering the current low awareness of gout prevention, screening, diagnosis, treatment and adherence to treatment in the young population globally, these results should draw attention from healthcare providers, physicians, parents, schools and those who are developing gout guidelines.

This analysis observed substantial increases in gout burden, regardless of the SDI, in the young population. This finding was different from the conclusion made in the GBD 2017 study conducted in all populations, suggesting that the growing gout burden was mainly in developed regions and countries.^18^ This difference in gout burden and SDI between analyses conducted in the young population and analyses conducted in the general population was also observed in children’s obesity and socioeconomic status (SES), and is probably explained by the fact that both the young population in low SES in developed countries and in high SES in developing countries had greater access to energy-dense diets.^23^ This study also confirmed that high BMI was the major contributor to YLD from gout in the young population, and its attributable risk has continued to increase in the past three decades for both males and females. The other risk factor for gout is kidney dysfunction, which contributed higher to gout in females than males. The above similarity and difference in risk factors for gout by gender can direct more detailed prevention campaigns for gout.

No region had decreasing trends in either gout incidence or YLD. The top two regions with the highest increase in both gout incidence and YLD simultaneously were high-income North America (including the USA, Canada and Greenland) and East Asia (including China, Taiwan Province of China and the Democratic People’s Republic of Korea). The rapidly increasing YLD and incidence rate indicated that these regions were highly unprepared for their rapidly growing gout burden in the young population. In 2019, while the young population in high-income North America was facing the highest rates of gout, their counterparts in East Asia were bearing the largest number of gout cases. However, high BMI contributed to nearly 50% of gout YLD in high-income America but contributed to only 20% of gout YLD in East Asia, indicating a need for different interventions for gout and definitions for high BMI in those two regions when designing future gout prevention campaigns.

Nationally, only 7% of the 204 countries had a decrease in gout incidence and YLD in the young population. The gout incidence in the young population varied largely from 19 to 124/100 000 population. Other than risk factors such as high BMI in North America, the national-level data further suggested a correlation between specific ethnic groups of certain islands and gout incidence. For example, Maldives and Taiwan (Province of China) had the third highest gout incidence in the young population. The high rates of gout among Taiwanese Aboriginals were reported previously.^24^ However, there was a lack of any gout studies in the Maldives.

For the first time, this study described and analysed the gout burden, its trends and risk factors in individuals aged 15–39 years at the global, regional and national levels. Our results advocate for more research on gout in younger populations and more representative data for each country and draw attention to the need for gout guidelines and gout intervention campaigns targeted at the young population. There are limitations when interpreting GBD data, although the GBD database has a rigorous modelling methodology. The results presented here were mainly derived from modelled data through processes in the Bayesian meta-regression tool DISMOD-MR 2.1, as only 36 countries with 130 data sources had national data on the prevalence and incidence of gout.^15^ The lack of data usually indicates a lack of healthcare resources, meaning the gout burden among adolescents and young adults probably was underestimated and underreported worldwide. The lack of data sources could also have introduced bias into our results. Hence, national-level estimates should be interpreted with caution.

Rapidly and substantially growing rates of gout in the young population are a public health issue globally in both developed and developing regions and countries. Improving representative national-level data on gout in younger populations, interventions for obesity, and increasing awareness about gout early detection and treatment in the young population are strongly suggested.

## Data Availability

Data are available upon reasonable request.
