# Supplementary material for: Global, regional and national burdens of gout in the young population from 1990 to 2019: a population-based study
Source: RMD Open. 2023 Apr 24;9(2):e003025. doi: 10.1136/rmdopen-2023-003025 (PMC10152042; doi:10.1136/rmdopen-2023-003025)
Supplement: Supplementary data [file rmdopen-2023-003025supp001.pdf]

Supplementary Materials

Supplement Data 1. Table 1 Incidence of gout and years living with gout disability and their average annual percentage changes from 1990 to 2019 in individuals aged 15–39 years at the global and regional levels.

Supplement Data 2. Overview, data sources, modeling, definition, and data process for Global Burden of Disease 2019.

Supplement Data 3. Gout case definition, input data, and methodology in Global Burden of Disease 2019.

Supplement Data 4. The prevalence, incidence, and years lived with disability rates of gout in adolescents and young adults aged 15-39 years from 1990 to 2019 at the global level.

Supplement Data 5. The incidence of gout in senior population aged over 55years from 1990 to 2019 at the global level.

Supplement Data 6. The joinpoint regression of gout incidence, prevalence, and YLD in adolescents and young adults aged 15-39 years between 1990 and 2019 globally.

Supplement Data 7. Rates and number of cases of gout by SDI.

Supplement Data 8. Proportional attributable fraction of high-BMI by sex, age subgroup, and regions.

Supplement Data 9. Proportional attributable fraction of kidney dysfunction by sex, age subgroup, and regions.

BMJ Publishing Group Limited (BMJ) disclaims all liability and responsibility arising from any reliance placed on this supplemental material which has been supplied by the author(s)

Supplement Data1. Table 1 Incidence of gout and years living with gout disability and their average annual percentage changes from 1990 to 2019 in individuals aged 15–39 years at the global and regional levels.

Table 1. Incidence of gout and years living with gout disability and their average annual percentage changes from 1990 to 2019 in individuals aged 15–39 years at the global and regional levels.

	Incidence (95% UI), per 100 000 population						YLD (95% UI), per 100 000 population					
	Cases in 1990	Rate in 1990	Cases in 2019	Rate in 2019	AAPC in rate, 1990–2019	P value	Cases in 1990	Rate in 1990	Cases in 2019	Rate in 2019	AAPC in rate, 1990–2019	P value
**Global**	849175 (594354–1172622)	38.71 (27.1–53.46)	1363085 (960539–1878303)	45.93 (32.36–63.29)	0.61 (0.57 to 0.65)	<0.001	107156 (63766–170053)	4.89 (2.91–7.75)	175377 (106554–271254)	5.91 (3.59–9.14)	0.66 (0.6 to 0.73)	<0.001
**Sex**												
Male	683214 (483447–943373)	61.57 (43.57–85.02)	1105822 (782548–1520040)	73.59 (52.08–101.16)	0.63 (0.58 to 0.68)	<0.001	85793 (51545–134495)	7.73 (4.65–12.12)	142072 (86213–216507)	9.45 (5.74–14.41)	0.69 (0.61 to 0.77)	<0.001
Female	165961 (110205–236278)	15.31 (10.17–21.8)	257263 (173316–366784)	17.56 (11.83–25.03)	0.51 (0.48 to 0.54)	<0.001	21362 (12165–34332)	1.97 (1.12–3.17)	33305 (19028–53566)	2.27 (1.3–3.66)	0.54 (0.51 to 0.58)	<0.001
**Age group**												
15–19	15075 (4326–31660)	2.9 (0.83–6.09)	18285 (5245–38107)	2.95 (0.85–6.15)	0.17 (0.11 to 0.22)	<0.001	754 (202–1716)	0.15 (0.04–0.33)	915 (246–2064)	0.15 (0.04–0.33)	0.17 (0.11 to 0.22)	<0.001
20–24	81467 (47825–122494)	16.54 (9.71–24.86)	101923 (60316–151331)	16.98 (10.05–25.22)	0.29 (0.21 to 0.36)	<0.001	6118 (2910–10869)	1.24 (0.59–2.21)	7600 (3684–13454)	1.27 (0.61–2.24)	0.24 (0.17 to 0.31)	<0.001
25–29	167398 (93456–263289)	37.8 (21.1–59.45)	246401 (140983–382268)	40.7 (23.28–63.14)	0.33 (0.28 to 0.37)	<0.001	18125 (9515–30088)	4.09 (2.15–6.79)	26654 (14458–43726)	4.4 (2.39–7.22)	0.33 (0.29 to 0.37)	<0.001
30–34	246281 (167701–342394)	63.86 (43.49–88.79)	427953 (294971–587577)	71.12 (49.02–97.65)	0.4 (0.35 to 0.45)	<0.001	32281 (17692–53260)	8.37 (4.59–13.81)	55995 (31044–92029)	9.31 (5.16–15.29)	0.4 (0.35 to 0.45)	<0.001
35–39	338953 (201952–540471)	96.09 (57.25–153.22)	568524 (337834–898443)	105.09 (62.45–166.08)	0.47 (0.4 to 0.55)	<0.001	49877 (29759–78857)	14.14 (8.44–22.36)	84213 (51315–132269)	15.57 (9.49–24.45)	0.5 (0.41 to 0.58)	<0.001
**SDI**												
High-middle SDI	172480 (120641–241383)	53.39 (37.34–74.72)	257269 (187860–342425)	77.65 (56.7–103.36)	1.28 (1.26 to 1.29)	<0.001	23554 (14172–37260)	7.29 (4.39–11.53)	36479 (23022–53809)	11.01 (6.95–16.24)	1.4 (1.35 to 1.44)	<0.001
High SDI	205498 (143456–285206)	42.5 (29.67–58.99)	297621 (207492–418459)	57.6 (40.16–80.98)	1.1 (1.03 to 1.17)	<0.001	25875 (14949–40534)	5.35 (3.09–8.38)	38266 (22455–60342)	7.41 (4.35–11.68)	1.15 (1.08 to 1.21)	<0.001

Low-middle SDI	299406 (209416–412802)	40.1 (28.05–55.29)	458145 (319253–635416)	48.99 (34.14–67.95)	0.71 (0.63 to 0.79)	<0.001	36968 (22192–58273)	4.95 (2.97–7.81)	57896 (34993–89400)	6.19 (3.74–9.56)	0.78 (0.67 to 0.9)	<0.001
Low SDI	123702 (86213–171580)	27.78 (19.36–38.53)	232070 (162104–321736)	31.56 (22.05–43.76)	0.39 (0.34 to 0.45)	<0.001	15002 (8765–23624)	3.37 (1.97–5.3)	28615 (16728–45126)	3.89 (2.27–6.14)	0.45 (0.38 to 0.52)	<0.001
Middle SDI	47724 (33241–66741)	24.62 (17.15–34.44)	117297 (81778–163192)	26.2 (18.27–36.45)	0.25 (0.23 to 0.26)	<0.001	5711 (3346–9041)	2.95 (1.73–4.66)	14035 (8215–22216)	3.13 (1.83–4.96)	0.27 (0.25 to 0.29)	<0.001
**Region**												
East Asia	3295 (2258–4674)	21.32 (14.61–30.25)	7571 (5265–10554)	29.48 (20.5–41.1)	1.13 (1.11 to 1.15)	<0.001	420 (227–680)	2.72 (1.47–4.4)	989 (544–1599)	3.85 (2.12–6.23)	1.2 (1.18 to 1.22)	<0.001
Oceania	4289 (3038–6039)	52.6 (37.25–74.06)	7080 (4959–9939)	72.86 (51.03–102.28)	1.01 (0.94 to 1.07)	<0.001	563 (333–921)	6.91 (4.08–11.29)	976 (574–1526)	10.04 (5.91–15.7)	1.21 (1.12 to 1.3)	<0.001
Central Asia	3103 (2114–4342)	20.91 (14.25–29.27)	4872 (3327–6787)	26.87 (18.35–37.44)	0.74 (0.63 to 0.85)	<0.001	403 (230–650)	2.72 (1.55–4.38)	640 (360–996)	3.53 (1.99–5.5)	0.75 (0.61 to 0.89)	<0.001
Central Europe	7743 (5320–10887)	27.19 (18.68–38.24)	12819 (8887–17852)	33.84 (23.46–47.12)	0.47 (0.3 to 0.65)	<0.001	957 (536–1536)	3.36 (1.88–5.39)	1597 (912–2568)	4.22 (2.41–6.78)	0.45 (0.26 to 0.64)	<0.001
Eastern Europe	10693 (7323–15092)	23.24 (15.91–32.79)	10248 (6962–14419)	28.78 (19.55–40.49)	0.94 (0.76 to 1.12)	<0.001	1359 (777–2167)	2.95 (1.69–4.71)	1310 (755–2099)	3.68 (2.12–5.89)	0.98 (0.77 to 1.18)	<0.001
High-income Asia Pacific	12196 (8275–17163)	17.88 (12.13–25.16)	23124 (15897–32196)	22.9 (15.74–31.88)	0.79 (0.75 to 0.83)	<0.001	1603 (912–2627)	2.35 (1.34–3.85)	3100 (1753–5006)	3.07 (1.74–4.96)	0.8 (0.73 to 0.87)	<0.001
Australasia	5440 (3767–7558)	26.2 (18.15–36.4)	14381 (9992–20055)	27.76 (19.29–38.72)	0.1 (0.06 to 0.14)	<0.001	645 (359–1037)	3.11 (1.73–5)	1718 (963–2765)	3.32 (1.86–5.34)	0.12 (0.08 to 0.17)	<0.001
Western Europe	314450 (222921–433376)	55.46 (39.32–76.44)	412946 (290224–575475)	80.07 (56.28–111.59)	1.36 (1.24 to 1.48)	<0.001	39352 (23515–62020)	6.94 (4.15–10.94)	53247 (31359–83469)	10.33 (6.08–16.19)	1.45 (1.28 to 1.62)	<0.001
Southern Latin America	27384 (18979–38220)	31.92 (22.12–44.55)	27015 (18607–38185)	39.36 (27.11–55.64)	0.59 (0.3 to 0.89)	<0.001	3386 (1963–5310)	3.95 (2.29–6.19)	3381 (1977–5381)	4.93 (2.88–7.84)	0.58 (0.25 to 0.92)	0.001
High-income North America	17518 (12175–24375)	24.85 (17.27–34.57)	45480 (31443–63051)	27.27 (18.86–37.81)	0.41 (0.35 to 0.46)	<0.001	2077 (1204–3283)	2.95 (1.71–4.66)	5425 (3168–8587)	3.25 (1.9–5.15)	0.45 (0.38 to 0.52)	<0.001
Caribbean	31379 (22144–44102)	46.46 (32.78–65.29)	30216 (21139–42581)	57.51 (40.23–81.04)	0.86 (0.74 to 0.98)	<0.001	4036 (2383–6307)	5.98 (3.53–9.34)	3976 (2370–6102)	7.57 (4.51–11.61)	0.99 (0.86 to 1.13)	<0.001

Andean Latin America	87514 (61078–123067)	77.4 (54.02–108.84)	149113 (111206–194327)	122.72 (91.52–159.93)	1.64 (1.58 to 1.7)	<0.001	12504 (7517–19832)	11.06 (6.65–17.54)	22057 (14186–31673)	18.15 (11.67–26.07)	1.74 (1.66 to 1.82)	<0.001
Central Latin America	39098 (27109–54377)	28.79 (19.97–40.05)	98905 (68300–138152)	38.24 (26.41–53.42)	0.99 (0.89 to 1.09)	<0.001	4749 (2748–7501)	3.5 (2.02–5.52)	12399 (7384–19493)	4.79 (2.86–7.54)	1.08 (0.97 to 1.19)	<0.001
Tropical Latin America	1254 (885–1745)	47.64 (33.62–66.3)	2918 (2066–4030)	53.62 (37.96–74.06)	0.29 (0.22 to 0.36)	<0.001	152 (86–241)	5.77 (3.27–9.14)	354 (202–568)	6.5 (3.72–10.43)	0.3 (0.24 to 0.37)	<0.001
North Africa and the Middle East	105440 (73307–147751)	24.41 (16.97–34.2)	211607 (146874–295561)	27.52 (19.1–38.44)	0.32 (0.28 to 0.36)	<0.001	12703 (7348–19950)	2.94 (1.7–4.62)	25773 (15197–41386)	3.35 (1.98–5.38)	0.36 (0.32 to 0.4)	<0.001
South Asia	158388 (109746–219542)	30 (20.79–41.58)	288868 (201696–402302)	34.04 (23.77–47.41)	0.37 (0.35 to 0.39)	<0.001	19204 (11459–30323)	3.64 (2.17–5.74)	35575 (21092–55877)	4.19 (2.49–6.58)	0.43 (0.4 to 0.45)	<0.001
Central Sub-Saharan Africa	9020 (6334–12719)	47.26 (33.19–66.64)	15678 (11141–22055)	61.65 (43.8–86.72)	0.82 (0.77 to 0.87)	<0.001	1190 (669–1889)	6.24 (3.51–9.9)	2091 (1226–3353)	8.22 (4.82–13.18)	0.9 (0.86 to 0.93)	<0.001
Eastern Sub-Saharan Africa	7054 (4929–9780)	32.17 (22.48–44.6)	13917 (9763–19415)	41.32 (28.99–57.64)	0.79 (0.66 to 0.91)	<0.001	840 (498–1349)	3.83 (2.27–6.15)	1689 (1001–2671)	5.01 (2.97–7.93)	0.83 (0.68 to 0.98)	<0.001
Southern Sub-Saharan Africa	13338 (9045–18590)	20.74 (14.06–28.9)	25614 (17734–35783)	28.75 (19.9–40.16)	1.17 (1.14 to 1.21)	<0.001	1705 (975–2720)	2.65 (1.52–4.23)	3334 (1882–5326)	3.74 (2.11–5.98)	1.19 (1.15 to 1.22)	<0.001
Western Sub-Saharan Africa	45565 (31815–65103)	31.63 (22.08–45.19)	48865 (33666–70080)	37.29 (25.69–53.48)	0.41 (0.27 to 0.54)	<0.001	5969 (3485–9550)	4.14 (2.42–6.63)	6564 (3865–10371)	5.01 (2.95–7.91)	0.51 (0.36 to 0.66)	<0.001
South-East Asia Region	19319 (13501–26872)	27.17 (18.99–37.79)	49244 (34119–68549)	27.49 (19.05–38.26)	0.03 (–0.04 to 0.09)	0.39	2317 (1361–3630)	3.26 (1.91–5.11)	5923 (3482–9338)	3.31 (1.94–5.21)	0.04 (–0.02 to 0.1)	0.18

Note: YLDs, years living with disability; UI, uncertainty intervals; NA, not available; AAPC, average annual percentage change; SDI, sociodemographic index.

Supplement Data 2. Overview, data source, and modeling for Global Burden of Disease 2019

Overview

The Global Burden of Disease (GBD) is an approach to global descriptive epidemiology.[1] It is a systematic, scientific effort to quantify the comparative magnitude of health loss due to diseases, injuries, and risk factors by age, sex, and geography for specific points in time. Institute for Health Metrics and Evaluation (IHME) serves as the coordinating center for the GBD and affiliated projects. Published in The Lancet in October 2020, GBD 2019 provides, for the first time, an independent estimation of population for each of 204 countries and territories and for the globe using a standardized, replicable approach, as well as a comprehensive update on fertility and migration.[1] GBD 2019 incorporates major data additions and improvements and methodological refinements. Mortality and life expectancy estimates have expanded to a total of 990 locations at the most detailed level, and new causes have been added to the fatal and nonfatal cause lists, for a total of 369 diseases and injuries (http://www.healthdata.org/gbd/about/protocol). GBD 2019 estimated each epidemiological quantity of interest—incidence, prevalence, mortality, years lived with disability (YLDs), years of life lost (YLLs), and disability-adjusted life-years (DALYs)—for 23 age groups; males, females, and both sexes combined; and 204 countries and territories that were grouped into 21 regions and seven super-regions. The GBD 2019 location hierarchy now includes all WHO member states. The GBD disease and injury analytical framework generated estimates for every year from 1990 to 2019. Diseases and injuries were organized into a levelled cause hierarchy from the three broadest causes of death and disability at Level 1 to the most specific causes at Level 4. Within the three Level 1 causes—communicable, maternal, neonatal, and nutritional diseases; noncommunicable diseases; and injuries—there are 22 Level 2 causes, 174 Level 3 causes, and 301 Level 4 causes (including 131 Level 3 causes that are not further disaggregated at Level 4). In total, 364 causes are nonfatal and 286 are fatal.[1]

Data sources

The GBD estimation process is based on identifying multiple relevant data sources for each disease or injury, including censuses, household surveys, civil registration and vital statistics, disease registries, health service use, air pollution monitors, satellite imaging, disease notifications, and other sources. Each of these types of data is identified from a systematic review of published studies, searches of government and international organization websites, published reports, primary data sources such as the Demographic and Health Surveys, and contributions of datasets by GBD collaborators. Aa total of 86,249 sources were used in this analysis, including 19,354 sources reporting deaths, 31,499 reporting incidence, 1973 reporting prevalence, and 26,631 reporting other metrics. Each newly identified and obtained data source is given a unique identifier by a team of librarians and included in the Global Health Data Exchange (GHDx). The GHDx makes publicly available the metadata for each source included in GBD as well as the data, where allowed by the data provider. Additional metadata for each source are available in the online GBD citation tool, http://ghdx.healthdata.org/gbd-results-tool.

Modeling

For most diseases and injuries, processed data are modeled using standardized tools to generate estimates of each quantity of interest by age, sex, location, and year.[1] There are three main standardized tools: the cause of death ensemble model (CODEm), spatiotemporal Gaussian process regression (ST-GPR), and DisMod-MR. Previous publications provide

more details on these general GBD methods.[2-4] Briefly, CODEm is a highly systematized tool to analyze cause of death data using an ensemble of different modeling methods for rates or cause fractions with varying choices of covariates that perform best with out-of-sample predictive validity testing. DisMod-MR is a Bayesian meta-regression tool that allows evaluation of all available data on incidence, prevalence, remission, and mortality for a disease, enforcing consistency between epidemiological parameters. Previous studies showed that DisMod-MR can produce robust and valid estimates compared with real surveillance data.[5] ST-GPR is a set of regression methods that borrow strength between locations and over time for single metrics of interest, such as risk factor exposure or mortality rates.[1]

Zhang J, *et al. RMD Open* 2023; 9:e003025. doi: 10.1136/rmdopen-2023-003025

Supplement Data 3. Gout case definition, input data, and methodology in Global Burden of Disease 2019.

Definition and data process

Gout was categorized under the musculoskeletal disorders in the Global Burden of Disease 2019. Gout is a rheumatic disease that is characterised by deposition of monosodium urate (MSU) crystals in the synovial fluid of joints and in other tissues, causing inflammation. The crystal formation is caused by elevated urate levels in extracellular fluids. GBD uses the case definition of primary gout given by the American College of Rheumatology, generally referred to as ARA 1977 survey criteria requiring the presence of MSU crystals in joint fluid or the presence of a tophus proven to contain MSU crystals and at least six of 12 gout symptoms or findings (>1 attack of acute arthritis, development of maximal inflammation within a day, attack of monoarticular arthritis, observation of joint erythema, pain or swelling in the first MTP joint, unilateral attack involving the first MTP joint, unilateral attack involving tarsal joint, suspected tophus, hyperuricemia, asymmetrical swelling within a joint on X-ray and negative culture of joint fluid for microorganisms during attack of joint inflammation) to make a diagnosis.

The ICD-10 code for gout is M10 and the ICD9 code is 274.

Flowchart

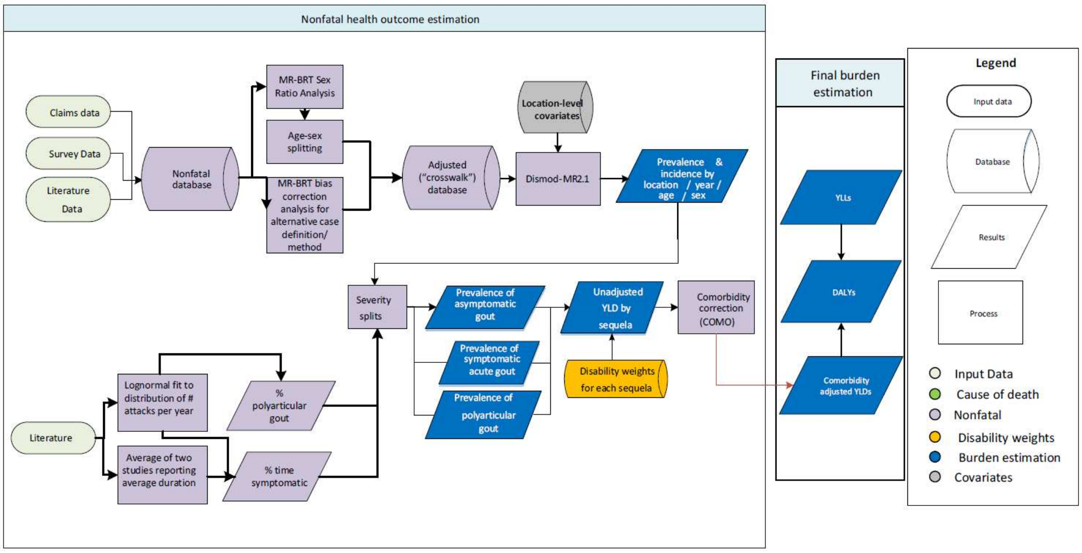

Input Data and Methodological Summary for Gout

Input data

The last systematic review was conducted in GBD 2013 for studies published between 1980 to 2009 using the following search terms on MEDLINE, EMBASE, CINAHL, CAB Abstracts, WHO Library (WHOLIS), and OpenSIGLE. For prevalence and incidence, the following search terms were used: (gout* OR hyperuricemia) AND (prevalen* OR inciden* OR cross-sectional OR cross sectional OR epidemiol* OR survey OR population-based OR population based OR population study OR population sample OR cohort OR follow-up OR follow up OR longitudinal OR regist*) AND (list of names of all GBD countries).

Exclusion criteria were:

• Sub-populations clearly not representative of the national population
• Not a population-based study
• Low sample size (less than 150)
• Review rather than original studies

For GBD 2019, 14 additional studies shared through the collaborator network were added. In addition, data from USA claims data for 2000 and 2010–2014 by state and Taiwan claims data from 2016 were included.

Data inputs for gout

Measure	Total sources	Countries with data
All measures	130	36
Prevalence	113	34
Incidence	15	6
Relative risk	3	2
Standardized mortality ratio	1	1
Proportion	7	3

Age and sex splitting

Reported estimates of prevalence were split by age and sex where possible. First, if studies reported prevalence for broad age groups by sex (eg, prevalence in 15- to 65-year-old males and females separately), and also by specific age groups for both sexes combined (eg, prevalence in 15- to 30-yearolds, then in 31- to 65-year-olds, for males and females combined), age-specific estimates were split by sex using the reported sex ratio and bounds of uncertainty. Second, prevalence data for both sexes that could not be split using a within-study ratio were split using a sex ratio derived from a meta-analysis of existing sex-specific data using MR-BRT. The female to male ratio was 0.33 (0.33 to 0.34). Finally, after the application of bias adjustments, where studies reported estimates across age groups 25 years or more, these were split into five-year age groups using the prevalence age pattern estimated by DisMod-MR 2.1 in GBD 2017.

Data adjustment

We used study covariates for studies relying on self-reported diagnoses and those identifying sources through a diagnostic code in administrative data, which include gout ICD codes as well as read codes used in the UK health system. We used MR-BRT to adjust alternative case definition and claims data in the USA from the year 2000 and from 2010 onward and for Taiwan claims data to the reference case definition. Matched data was based off of age, sex, year, and location. The mean and standard error for the coefficients were calculated using the MR-BRT crosswalk adjustment method. Betas and exponentiated values (which can be interpreted as an odds ratio) for these covariates are shown in the table below:

MR-BRT crosswalk adjustment factors for gout

Data input	Reference or alternative case definition	Gamma	Beta Coefficient, Log (95% CI)	Adjustment factor*
Physician-diagnosed gout	Ref	0.55	--	--
Self-reported gout	Alt		0.33 (0.050 to 0.60)	1.30 (1.05 to 1.83)
Gout identified with administrative data	Alt		0.29 (0.29 to 0.30)	1.34 (1.34 to 1.35)

8

USA claims data-2000	Alt		-1.88 (-2.84 to -0.92)	0.15 (0.058 to 0.40)
USA claims data-2010-2016	Alt		-1.55 (-2.00 to -1.09)	0.22 (0.13 to 0.34)
Taiwan claims data-2016	Alt		0.30 (0.27 to 0.33)	1.35 (1.31 to 1.40)

*Adjustment factor is the transformed Beta coefficient in normal space, and can be interpreted as the factor by which the alternative case definition is adjusted to reflect what it would have been if measured as the reference.

Modeling strategy

Prior settings included assuming the excess mortality rate and remission of gout did not exceed 0.01 and 0.2, respectively, and that there was no incidence or prevalence of gout before the age of 15 years. We have made no substantive changes in the modeling strategy from GBD 2017, with the exception of increasing the coefficient of variation from 0.4 at the Global, Super Region, and Region levels to 0.8 to allow the model to better follow the data. We included the summary exposure variable (SEV) scalar for gout which summarises exposure to risks estimated in GBD to impinge on gout, ie, low glomerular filtration rate, as a country covariate. We set bounds of 0.75 to 1.25 as the SEV is constructed in a way that if our risk estimates are accurate the value should be 1.

Severity and Disability

The basis of the GBD disability weight (DW) survey assessments are lay descriptions of sequelae highlighting major functional consequences and symptoms. The lay descriptions and disability weights for gout severity levels are shown below.

Severity distribution, details on the severity levels for gout in GBD 2019 and the associated disability weight (DW) with that severity.

Severity level	Lay description	DW (95% CI)
Gout, acute	This person has severe pain and swelling in the leg, making it very difficult to get up and down, stand, walk, lift, and carry heavy things. The person has trouble sleeping because of the pain.	0.295 (0.196-0.409)
Polyarticular gout (same as for severe RA)	This person has severe, constant pain and deformity in most joints, causing difficulty moving around, getting up and down, eating, dressing, lifting, carrying, and using the hands. The person often feels sadness, anxiety, and extreme fatigue.	0.581 (0.403-0.739)
Asymptomatic gout	This person has a diagnosis of gout without pain or functional difficulties	0

To calculate the severity distribution of gout, we used three studies on the distribution of the

number of gout attacks per year and fitted a lognormal curve using a least squared differences method. In the absence of data on the proportion of gout cases who have chronic polyarticular gout, we assumed the proportion is equal to those who would have 52 attacks a year (ie, weekly) or more as implied by the lognormal curve.

The average number of attacks was estimated from the lognormal fit: 5.66 (5.14–6.18). From two studies we derived an average duration of attacks of 6.1 (5.4–6.8) days by simple averaging. The resulting proportion of time symptomatic for acute gout was taken as the multiplication of these two estimates divided by the number of days in a year: 9.4% (8.0–10.9%).

References

1. Edwards NL, Sundy JS, Forsythe A, Blume S, Pan F, Becker MA. Work productivity loss due to flares in patients with chronic gout refractory to conventional therapy. Journal of Medical Economics. 2011; 14(1).
2. Yu, KH, Luo SF, et al. Younger age of onset of gout in Taiwan. Rheumatology. 2003; 42(1): p. 166-170.
3. Yu TF, et al. Diversity of clinical features in gouty arthritis. Seminars in Arthritis and Rheumatism. 1984; 13(4): p. 360-368.

Supplement Data 4. The prevalence, incidence, and years lived with disability rates of gout in adolescents and young adults aged 15-39 years from 1990 to 2019 at the global level.

Year	gout, rate (95% UI) per 100000 population					
	Prevalence	95% CI	Incidence	95% CI	YLD	95% CI
1990	145.19	100.2 to 202.76	38.71	1990 to 38.71	4.89	2.91 to 7.75
1991	146.01	100.76 to 203.47	38.88	1991 to 38.88	4.92	2.93 to 7.68
1992	146.93	101.4 to 204.43	39.07	1992 to 39.07	4.94	2.96 to 7.75
1993	147.82	102.01 to 205.47	39.26	1993 to 39.26	4.98	2.99 to 7.75
1994	148.65	102.57 to 206.64	39.44	1994 to 39.44	5.01	3.02 to 7.78
1995	149.53	103.13 to 208.07	39.63	1995 to 39.63	5.04	3.03 to 7.86
1996	150.73	104.03 to 209.54	39.89	1996 to 39.89	5.08	3.05 to 7.9
1997	152.44	105.31 to 212.18	40.26	1997 to 40.26	5.13	3.09 to 7.98
1998	154.64	106.97 to 215.32	40.73	1998 to 40.73	5.21	3.13 to 8.13
1999	157.02	108.8 to 218.14	41.22	1999 to 41.22	5.28	3.16 to 8.18
2000	159.13	110.44 to 220.79	41.65	2000 to 41.65	5.36	3.22 to 8.31
2001	161.04	111.48 to 223.52	42.03	2001 to 42.03	5.42	3.28 to 8.37
2002	162.89	112.7 to 226.18	42.43	2002 to 42.43	5.48	3.33 to 8.54
2003	164.29	114.27 to 228.01	42.74	2003 to 42.74	5.53	3.33 to 8.52
2004	165.18	115.26 to 229.13	42.94	2004 to 42.94	5.56	3.34 to 8.61
2005	165.53	115.74 to 229.38	43.01	2005 to 43.01	5.57	3.36 to 8.67
2006	165.84	116.37 to 229.09	43.08	2006 to 43.08	5.59	3.39 to 8.57
2007	166.29	117.16 to 229.01	43.18	2007 to 43.18	5.60	3.4 to 8.59
2008	166.75	117.94 to 229.56	43.30	2008 to 43.3	5.61	3.39 to 8.63
2009	167.05	118.54 to 229.83	43.42	2009 to 43.42	5.62	3.41 to 8.65
2010	167.12	118.83 to 229.4	43.53	2010 to 43.53	5.63	3.41 to 8.61
2011	167.08	118.75 to 229.31	43.66	2011 to 43.66	5.63	3.42 to 8.65
2012	167.32	118.87 to 229.65	43.85	2012 to 43.85	5.64	3.42 to 8.6
2013	167.93	119.28 to 230.56	44.12	2013 to 44.12	5.66	3.44 to 8.69
2014	168.95	119.96 to 232.05	44.45	2014 to 44.45	5.69	3.48 to 8.81
2015	170.38	121.02 to 234.02	44.83	2015 to 44.83	5.74	3.49 to 8.83
2016	173.49	123.57 to 238.15	45.56	2016 to 45.56	5.84	3.57 to 8.98
2017	176.43	125.91 to 242.96	46.21	2017 to 46.21	5.93	3.62 to 9.05
2018	176.93	125.55 to 243.63	46.27	2018 to 46.27	5.95	3.6 to 9.16
2019	175.68	123.78 to 242.53	45.93	2019 to 45.93	5.91	3.59 to 9.14

Supplement Data 5. The incidence of gout in senior population aged over 55years from 1990 to 2019 at the global level.

Year	Incidence rate per 100 000 population	95% CI lower	95% CI upper
1990	307.2856808	410.308	225.8532
1991	305.9773958	408.5006	225.3041
1992	304.765622	406.7928	224.8023
1993	303.8665672	405.4772	224.5249
1994	303.4752208	404.8217	224.6847
1995	303.6833375	404.1583	225.3792
1996	304.3042593	404.3793	225.6123
1997	305.020984	404.7103	225.9555
1998	305.942931	405.2992	226.8294
1999	306.9976559	406.0227	227.4356
2000	308.2647622	407.39	228.0862
2001	310.5946462	410.6131	229.9605
2002	314.2873827	415.6476	232.8822
2003	318.763158	422.0685	236.4508
2004	323.2269755	428.3885	240.1688
2005	327.1192055	433.5564	243.5506
2006	331.701776	439.955	246.8597
2007	338.600849	448.4973	251.9806
2008	345.9987421	457.4768	258.3688
2009	352.4814948	465.6196	264.1286
2010	356.2992934	470.6868	267.9168
2011	358.2367032	473.3613	269.526
2012	359.877649	475.2542	270.645
2013	361.5877114	477.4505	271.7524
2014	364.0229702	480.6621	273.783
2015	367.5399924	485.5992	276.3855
2016	377.0283618	497.2748	282.6505
2017	384.9452385	507.489	288.5724
2018	383.9028046	506.2817	287.7675
2019	377.4045539	498.0329	282.5615

The AAPC of the incidence rate of gout and 95% CI during 1990 and 2019 was calculated. AAPC= 0.93 (95% CI: 0.83 to 1.03)

Zhang J, *et al. RMD Open* 2023; 9:e003025. doi: 10.1136/rmdopen-2023-003025

Supplement Data 6. The joinpoint regression of gout incidence, prevalence, and YLD in adolescents and young adults aged 15-39 years between 1990 and 2019 globally.

Lower Endpoint	Upper Endpoint	APC	Lower CI	Upper CI	Test Statistic (t)	Prob > |t|
**YLDs**						
1990	1996	0.6*	0.6	0.7	21.8	< 0.001
1996	2003	1.3*	1.2	1.3	43.7	< 0.001
2003	2014	0.2*	0.2	0.2	14.4	< 0.001
2014	2017	1.5*	1.1	1.9	8.7	< 0.001
2017	2019	-0.1	-0.5	0.3	-0.5	0.592
**Incidence**						
1990	1996	0.5*	0.5	0.6	27.3	< 0.001
1996	2003	1.0*	1	1.1	53.9	< 0.001
2003	2011	0.2*	0.2	0.3	15.1	< 0.001
2011	2014	0.6*	0.3	0.8	5	< 0.001
2014	2017	1.4*	1.1	1.6	12.3	< 0.001
2017	2019	-0.2	-0.5	0	-2.1	0.059
**Prevalence**						
1990	1996	0.6*	0.6	0.7	22	< 0.001
1996	2003	1.3*	1.2	1.4	44.9	< 0.001
2003	2014	0.2*	0.2	0.2	13.6	< 0.001
2014	2017	1.6*	1.2	2	9.3	< 0.001
2017	2019	-0.1	-0.5	0.2	-0.7	0.493

Zhang J, *et al. RMD Open* 2023; 9:e003025. doi: 10.1136/rmdopen-2023-003025

Supplement Data 7. Rates and number of cases of gout by SDI.

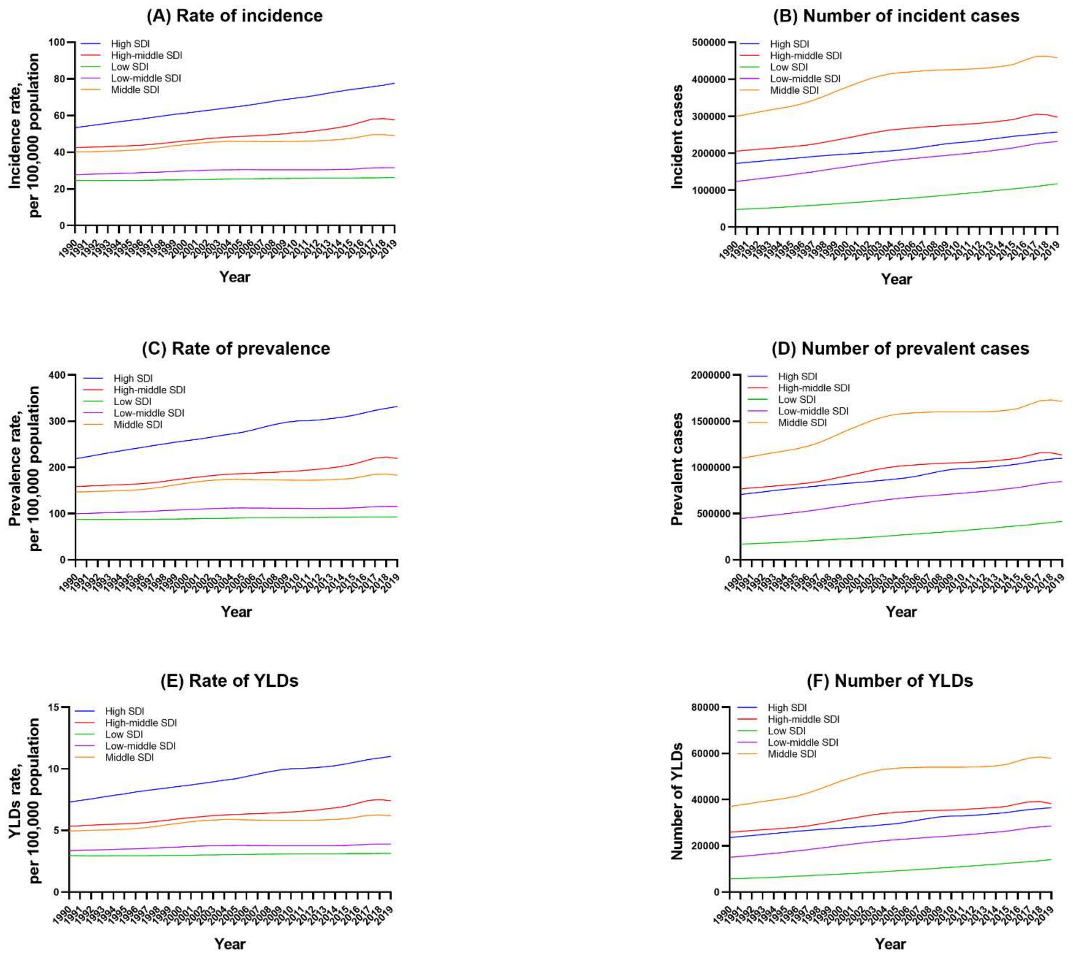

Supplement 8. Proportional attributable fraction of high-BMI by sex, age subgroup, and regions.

	Proportional attributable fractions	Lower 95% CI	Higher 95% CI
**Total**			
**Age subgroups**			
15-19	--	--	--
20-24	20.17%	9.85%	34.31%
25-29	26.13%	13.67%	42.49%
30-34	29.36%	15.82%	46.41%
35-39	32.03%	17.68%	50.11%
**Sex**			
Male	30.69%	15.56%	49.10%
Female	24.88%	13.55%	38.49%
**Regions**			
Andean Latin America	38.72%	22.19%	57.70%
Australasia	46.08%	27.32%	67.31%
Caribbean	34.39%	20.53%	51.10%
Central Asia	36.73%	20.95%	55.62%
Central Europe	39.95%	23.66%	58.92%
Central Latin America	38.02%	22.95%	55.49%
Central Sub-Saharan Africa	15.41%	6.49%	28.34%
East Asia	22.67%	9.62%	40.27%
Eastern Europe	37.54%	21.99%	56.09%
Eastern Mediterranean Region	37.62%	22.55%	55.10%
Eastern Sub-Saharan Africa	20.13%	9.87%	34.52%
High-income Asia Pacific	19.68%	7.63%	37.52%
High-income North America	49.92%	28.93%	70.93%
North Africa and Middle East	43.31%	26.60%	61.69%
Oceania	26.13%	12.79%	43.57%
South Asia	20.99%	10.47%	35.81%
Southeast Asia	24.14%	12.79%	39.43%
Southern Latin America	36.36%	18.69%	57.38%
Southern Sub-Saharan Africa	35.05%	20.38%	52.30%
Tropical Latin America	40.97%	25.44%	58.28%
Western Europe	35.07%	18.63%	54.75%
Western Pacific Region	22.94%	10.13%	40.42%
Western Sub-Saharan Africa	26.47%	14.04%	42.13%

Supplement 9. Proportional attributable fraction of kidney dysfunction by sex, age subgroup, and regions.

	Proportional attributable fractions	Lower 95% CI	Higher 95% CI
**Total**			
**Age subgroups**			
15-19	--	--	--
20-24	--	--	--
25-29	1.23%	1.00%	1.48%
30-34	1.38%	1.13%	1.67%
35-39	1.71%	1.38%	2.09%
**Sex**			
Male	1.41%	1.18%	1.66%
Female	1.61%	1.34%	1.92%
**Regions**			
Andean Latin America	1.69%	1.41%	2.02%
Australasia	0.80%	0.64%	1.00%
Caribbean	2.03%	1.68%	2.43%
Central Asia	2.97%	2.48%	3.53%
Central Europe	1.40%	1.11%	1.71%
Central Latin America	2.88%	2.41%	3.44%
Central Sub-Saharan Africa	1.01%	0.83%	1.21%
East Asia	1.07%	0.88%	1.27%
Eastern Europe	2.72%	2.23%	3.31%
Eastern Sub-Saharan Africa	0.95%	0.79%	1.12%
High-income Asia Pacific	1.05%	0.84%	1.30%
High-income North America	1.36%	1.12%	1.62%
North Africa and Middle East	2.14%	1.79%	2.52%
Oceania	1.81%	1.52%	2.14%
South Asia	1.51%	1.27%	1.80%
Southeast Asia	1.91%	1.62%	2.25%
South-East Asia Region	1.64%	1.38%	1.94%
Southern Latin America	1.02%	0.83%	1.24%
Southern Sub-Saharan Africa	1.84%	1.52%	2.21%
Tropical Latin America	1.72%	1.40%	2.06%
Western Europe	0.69%	0.55%	0.85%
Western Sub-Saharan Africa	1.68%	1.41%	1.98%

Zhang J, *et al. RMD Open* 2023; 9:e003025. doi: 10.1136/rmdopen-2023-003025
